# Acquired immunodeficiency syndrome-related progressive multifocal leukoencephalopathy-immune reconstitution inflammatory syndrome: prevalence, main characteristics, and outcomes in a Brazilian center

**DOI:** 10.1055/s-0043-1772831

**Published:** 2023-10-29

**Authors:** Monize Nascimento Santana, Raphaela Ferrari, Arthur Cassa Macedo, Rosa Maria Nascimento Marcusso, Ruan de Andrade Fernandes, José Ernesto Vidal

**Affiliations:** 1 Instituto de Infectologia Emílio Ribas, Departamento de Infectologia, São Paulo SP, Brazil.; 2 McGill University, Department of Neurology and Neurosurgery, Montreal QC, Canada.; 3 Instituto de Infectologia Emílio Ribas, Departamento de Neurologia, São Paulo SP, Brazil.; 4 Universidade de São Paulo, Faculdade de Medicina, Hospital das Clínicas, Departamento de Moléstias Infecciosas e Parasitárias, São Paulo SP, Brazil.; 5 Universidade de São Paulo, Faculdade de Medicina, Laboratório de Investigação Médica (LIM 49), São Paulo SP, Brazil.

**Keywords:** Leukoencephalopathy, Progressive Multifocal, Immune Reconstitution Inflammatory Syndrome, Epidemiology, HIV, Brazil, Leucoencefalopatia Multifocal Progressiva, Síndrome Inflamatória da Reconstituição Imune, Epidemiologia, HIV, Brasil

## Abstract

**Background** Progressive multifocal leukoencephalopathy (PML) - immune reconstitution inflammatory syndrome (IRIS) in people living with HIV/AIDS (PLWHA) has been rarely described in low- and middle-income countries.

**Objective** To describe the prevalence of PML-IRIS among PLWHA with PML and its main features in a tertiary hospital in Brazil.

**Methods** We performed a retrospective cohort study. We included PLWHA with PML-IRIS patients admitted at *Instituto de Infectologia Emílio Ribas* , São Paulo, Brazil, between 2011 and 2021. We retrieved information on neurological manifestations, neuroimaging findings, treatments, and outcomes.

**Results** We identified 11 (11.8%) PML-IRIS cases among 93 patients with definite PML. Eight (73%) cases were men and had a median (IQR) age of 41 (27–50) years. Seven (63.6%) patients developed unmasking PML-IRIS and 4 (36.4%) had paradoxical PML-IRIS. The median (IQR) time from initiation of combined antiretroviral therapy (cART) to IRIS diagnosis was 49 (30–70) days. Ten (90.9%) patients received corticosteroids. There were 4 (36%) in-hospital deaths and 3 were associated with hospital-acquired pneumonia. Among the 7 (64%) patients who survived, 5 (71.5%) had sequelae at discharge. One year after the PML-IRIS diagnosis, 6 (54.5%) patients were alive.

**Conclusion** The prevalence of PML-IRIS was 11.8%. Most patients had unmasking PML-IRIS. In-hospital mortality and morbidity were high. One-year survival was similar to that described in some high-income countries.

## Resumo

**Antecedentes** A síndrome inflamatória de reconstituição imune (SIRI) da leucoencefalopatia multifocal progressiva (LEMP) em pessoas vivendo com HIV/Aids (PVHA) foi raramente descrita em países de baixa e média renda.

**Objetivo** Descrever a prevalência da SIRI-LEMP- em PVHA com LEMP e suas principais características em um hospital no Brasil.

**Métodos** Foi realizado um estudo de coorte retrospectivo. Incluímos PVHA com SIRI-LEMP admitidos no Instituto de Infectologia Emílio Ribas, São Paulo, Brasil, entre 2011 e 2021. Recuperamos informações sobre manifestações neurológicas, neuroimagem, tratamento e desfecho.

**Resultados** Identificamos 11 (11,8%) casos de SIRI-LEMP entre 93 pacientes com LEMP definitiva. Oito (73%) casos eram homens e a mediana de idade (amplitude interquartile - AIQ) foi de 41 (27–50) anos. Sete (63,6%) pacientes desenvolveram SIRI-LEMP “desmascarada” e 4 (36,4%) casos apresentaram SIRI-LEMP “paradoxal”. A mediana de tempo (AIQ) desde o início da terapia antirretroviral combinada (cART) até o diagnóstico de SIRI foi de 49 (30–70) dias. Dez (90,9%) pacientes receberam corticoide. Houve 4 (36%) óbitos intra-hospitalares e 3 foram associados à pneumonia hospitalar. Dos 7 (64%) pacientes que sobreviveram, 5 (71,5%) ficaram com sequelas na alta. Um ano após o diagnóstico de SIRI-LEMP, 6 (54,5%) pacientes estavam vivos.

**Conclusão** A prevalência de SIRI-LEMP foi de 11,8%. A maioria dos pacientes apresentava SIRI-LEMP “desmascarada”. A mortalidade e morbidade hospitalar foram altas. A sobrevida em 1 ano foi semelhante à descrita em alguns países de alta renda.

## INTRODUCTION

Progressive multifocal leukoencephalopathy (PML) is a demyelinating brain disease caused by the John Cunningham virus (JCV). 1 2 It was first described in 1958 by Åström et al. 3 and finally associated to the JCV in 1971 by Padgett et al. 4

Before the human immunodeficiency virus (HIV) epidemic, PML was recognized as a very rare and fatal complication of either hematological malignancies or systemic inflammatory disorders. A literature review between 1958 and 1982 reported only 230 PML cases. 5 In the precombined antiretroviral therapy (cART) era, HIV has become the most important underlying cause of immunosuppression in patients with PML. Previous studies indicate that 3 to 5% of people living with HIV/AIDS (PLWHA) present with PML, 6 with a 1-year survival rate of 10%. 7

In the cART era, an important incidence decrease of HIV-related PML has been described, even though this reduction was lower than in other opportunistic diseases. 8 9 In addition, the 1-year survival rate has increased to approximately 50% in PLWHA cases when using cART. 10 11

Despite the benefits of cART in the immune function and outcomes of PLWHA with PML, its use may cause an aberrant inflammatory response 12 named immune reconstitution inflammatory syndrome (IRIS). 13 Progressive multifocal leukoencephalopathy-associated IRIS can be developed in two different settings: worsening of previously diagnosed PML after cART (paradoxical PML-IRIS) and *de novo* PML after initiation of cART (unmasking PML-IRIS). 14 A systematic review and meta-analysis reported that 3 (16.7%) out of 52 HIV-related PML patients had IRIS and were classified as paradoxical IRIS. 15 Another systematic review identified 46 cases of IRIS in HIV-related PML (21 unmasking PML-IRIS and 25 paradoxical PML-IRIS), 14 including 1 from Brazil 16 and 1 from Mexico. 17

There is scarce information about PML-IRIS in PLWHA from low- and middle-income countries. In this study, we sought to estimate the prevalence of PML-IRIS among PLWHA with definite PML as well as to describe its main features and outcomes in a tertiary center in São Paulo, Brazil.

## METHODS

We performed a retrospective cohort study including PLWHA patients with PML-related IRIS admitted at the *Instituto de Infectologia Emílio Ribas* , located in São Paulo, Brazil, between January 2011 and December 2021.

The inclusion criteria were participants with:

- confirmed HIV infection;
- definite PML (i.e., presence of compatible clinical and neuroradiology features associated with the detection of JCV DNA in the cerebrospinal fluid [CSF]);
- treatment with cART resulting in a decrease in plasma HIV viral load;
- symptoms consistent with an infectious or inflammatory condition that appeared while the patient was being treated with cART;
- symptoms that could not be explained by a new acquired infection, the expected course of a newly diagnosed opportunistic infection, or drug toxicity. 18 19 20

PML-IRIS was classified as:

- unmasking, if the patient presented new onset neurological manifestations; or
- paradoxical, if neurological manifestations were exacerbated after the initiation of cART. 18 19 20

Potential participants were retrieved from the databases of the *Instituto de Medicina Tropical de São Paulo* and the *Instituto Adolfo Lutz* , which were the reference laboratory centers in São Paulo where the qualitative CSF JCV-polymerase chain reaction (PCR) testing was performed.

Then, we screened the medical records of patients with detectable JCV in the CSF to identify cases fulfilling the criteria for definite PML. Finally, we selected the cases with PML-IRIS and obtained their demographic information as well as data regarding clinical presentation, neuroimaging findings, treatments, and clinical outcomes. All data were registered in a standardized electronic form.

Data for continuous variables were described with median and interquartile range (IQR), and for categorical variables as frequency and percentages. This study was designed and reported according to the Strengthening the Reporting of Observational Studies in Epidemiology (STROBE) guidelines.

The present study was approved by the Scientific Division and the Ethics Committee of *Instituto de Infectologia Emílio Ribas* (Protocol Number 33/2022).

## RESULTS

During the study period, 14,490 PLWHA patients were admitted to our institution, 93 (0.6%) of them fulfilled the criteria for definite PML, and 11 (0.08%) had PML-IRIS. Among the cases with PML, 11 (11.8%) had PML-IRIS and were included in the present study. Their median (interquartile - IQR) age was 41 (27–50) years, and 8 (73%) participants were men. Seven (63.6%) patients presented with unmasking PML-IRIS and 4 (36.4%) with paradoxical PML-IRIS. Table 1 shows the main findings and outcomes of the patients included in this study, and Table 2 shows their main individual characteristics.

**Table 1:** Main findings and outcomes of 11 people living with HIV/AIDS with PML-IRIS admitted at *Instituto de Infectologia Emilio Ribas* between 2011 and 2021

Variables		Value
Demographic data	Age, years, median (IQR)	41 (27–50)
	Male, *n* (%)	8 (73)
	Time between cART and PML-IRIS, days, median (IQR)	49 (30–70)
Clinical features	Motor deficits, *n* (%)	8 (72.7)
	Gait disturbance, *n* (%)	7 (63.6)
	Speech disorders, *n* (%)	6 (54.5)
	Mental confusion, *n* (%)	6 (54.5)
	Cerebellar ataxia, *n* (%)	3 (27.3)
	Seizure, *n* (%)	3 (27.3)
	Visual disturbances, *n* (%)	1 (9.1)
Brain MRI abnormalities, *n* = 9	Hypointense lesions on T1-weighted and hyperintense lesions on T2-weighted and FLAIR sequences, *n* (%)	9 (100)
	Contrast-enhanced lesions, *n* ^a^ (%)	3 (37.5)
	Mass effect, *n* (%)	1 (11)
Laboratory features	HIV VL before cART, copies/mL, median (IQR)	9,005 (1,772–98,358)
	HIV VL at PML-IRIS diagnosis, copies/mL, median (IQR)	67 (0–101)
	CD4 T cell count before cART, cells/μl, median (IQR)	66 (22–91)
	CD4 T cell count at PML-IRIS diagnosis, cells/μl, median (IQR)	50 (22–53)
Proposed treatment during hospital admission	Maintaining cART, *n* (%)	11 (100)
	Corticosteroid, *n* (%)	10 (90.3)
Clinical outcome	In-hospital survival, *n* (%)	7 (63.4)
	Neurological sequelae at hospital discharge, *n* (%)	5 (71.5)
	One-year survival, *n* (%)	6 (54.5)
	Two-year survival, *n* (%)	6 (54.5)

Abbreviations: cART, combined antiretroviral therapy; HIV, human immunodeficiency virus; IQR, interquartile range; MRI, magnetic resonance imaging; PML-IRIS, progressive multifocal leukoencephalopathy-immune reconstitution inflammatory syndrome; VL, viral load;.

Note: ^a^ Proportion calculated out of 8 patients since one patient had a contraindication to the use of contrast (acute kidney failure).

**Table 2:** Main individual characteristics of the 11 people living with HIV/AIDS with PML-IRIS admitted at *Instituto de Infectologia Emilio Ribas* between 2011 and 2021

Case	Age/ Sex	HIV VL before cART copies/mL (log)	HIV VL at PML-IRIS onset copies/mL (log)	CD4+ before cART (cells/µL)	CD4+ at PML-IRIS onset (cells/µL)	Delay between cART and PML-IRIS (days)	Corticosteroid initial dose / Length of treatment	cART	Admission duration (days)	Category of PML-IRIS	In-hospital outcome (cause of death)
1	44/M	9,005 (3.9)	60 (1.7)	77	22	31	Dexamethasone 10 mg/day, IV /1 day	AZT/3TC + EFV	74	Paradoxical	Death (unknown)
2	45/F	1,772 (3.2)	425 (2.6)	22	53	70	Hydrocortisone 300 mg/day, IV / 13 days	AZT/3TC + EFV	80	Unmasking	Discharge
3	35/M	12,488 (4.19)	74 (1.8)	31	52	64	–	AZT/3TC + LPV/r	13	Unmasking	Discharge
4	53/M	5,387 (3.7)	89 (1.9)	125	23	8	Dexamethasone 16 mg/day, EFT /18 days	AZT/3TC + EFV	54	Unmasking	Death (in-hospital pneumonia)
5	38/F	667 (2.8)	67 (1.8)	117	98	49	Dexamethasone 16 mg/day, IV /3 days	TDF/3TC/EFV	4	Unmasking	Discharge
6	63/M	225 (2.3)	0	91	50	42	Dexamethasone 16 mg/day, IV /11 days	AZT/3TC + LPV/r	48	Paradoxical	Death (in-hospital pneumonia)
7	25/M	402,568 (5.6)	612 (2.7)	30	146	68	Dexamethasone 16 mg/day, IV /10 days	TDF/3TC + ATV/r	11	Unmasking	Death (in-hospital pneumonia)
8	27/M	30,431 (4.5)	0	8	53	103	Prednisone 20 mg/day, PO /5 days	TDF/3TC/EFV	11	Unmasking	Discharge
9	20/M	5,000 (3.5)	0	66	22	458	Dexamethasone 16 mg/day, IV /8 days	ABC + 3TC+ DTG + ATV/r	37	Unmasking	Discharge
10	41/M	98,358 (4.9)	0	80	44	28	Dexamethasone 16 mg/day, IV /21 days	AZT/3TC + LPV/r	14	Paradoxical	Discharge
11	50/F	136,856 (5.1)	101 (2.0)	10	19	30	Hydrocortisone 300 mg/day, IV /5 days	TDF/3TC + DTG	29	Paradoxical	Discharge

Abbreviations: 3TC, lamivudine; ATV, atazanavir; AZT, zidovudine; cART, combined antiretroviral therapy; DTG, dolutegravir; EFT, enteral feeding tube; EFV, efavirenz; IV, intravenous; LPV, lopinavir; PML-IRIS, progressive multifocal leukoencephalopathy-immune restoration inflammatory syndrome; PO, oral administration; r, ritonavir; TDF, tenofovir disoproxil fumarate; VL, viral load.

All patients were aware of their HIV status before admission. The median (IQR) time of HIV diagnosis was 96 (3–240) months. Seven (64%) patients had a previous AIDS-defining illness. Eight (73%) were on regular use of cART at admission, with 5 (45.5%) of them being in use of a protease inhibitor. The median (IQR) time from the initiation of cART to the IRIS diagnosis was 49 (30–70) days. Their main neurological manifestations were motor deficit (n = 8; 73%), gait disturbance (n = 7; 63%), speech disorders (n = 6; 54.5%), and confusion (n = 6; 54.5%) ( Table 1 ).

The median (IQR) of CD4+ T-cell count before cART was 66 (22–91) cells/μL and, at the time of PML-IRIS diagnosis, it was 50 (22–53) cells/μL. The median (IQR) of HIV viral load before cART was 9,005 (1,772–98,358) copies/mL and, at the time of PML-IRIS diagnosis, it was 67 (0–101) copies/mL ( Table 1 ). At diagnosis, 4 (36.4%) cases had ≤ 50 copies/mL and 9 (81.8%) had ≤ 200 copies/mL. In the CSF, the total leukocyte count median (IQR) was 1 cell/mm ^3^ (0–2), while the median (IQR) glucose and protein levels were, respectively, 61 mg/dL (50–69) and 36 mg/dL (25–41).

Magnetic resonance imaging (MRI) was performed in 9 (82%) patients. All of them presented hypointense lesions on T1-weighted and hyperintense lesions on T2-weighted and fluid-attenuated inversion recovery (FLAIR) sequences. Eight (89%) patients had multiple lesions on magnetic resonance imaging (MRI). Three (37.5%) of 8 patients presented contrast-enhanced lesions (cases 6, 9, and 11; Table 2 ) and 1 (11%) patient (case 2) presented mass effect. Two (22%) patients had hypointense lesions on T1-weighted and hyperintense lesions on T2-weighted and FLAIR sequences only in the cerebellum (cases 7 and 9), while 5 (55.5%) presented cortical atrophy (cases 2, 3, 6, 7, and 11). Nine (82%) patients underwent computed tomography (CT), 3 (33%) of which presented mass effect (cases 2, 4, and 5; Table 2 ). Two (28.5%) of the 7 patients who had contrast injections presented contrast-enhanced lesions (cases 4 and 9). Computed tomography was the single neuroimaging in 2 patients: one (case 4) presented a single hypodense lesion in the cerebellum with mass effect, without contrast-enhanced lesions or cortical atrophy, and the other (case 5) multiple hypodense lesions and cortical atrophy, without mass effect or contrast-enhanced lesions.

During hospitalization, 10 (90.9%) patients received corticosteroids and all of them maintained the use of cART. The median (IQR) length of hospital stay was 29 (11–54) days. There were 4 (36%) in-hospital deaths, 3 due to hospital-acquired pneumonia, and 1 to an unknown cause. Among the 7 (64%) patients who survived, 5 (71.5%) had sequelae at discharge. One year after the PML-IRIS diagnosis, 6 (54.5%) patients were alive and had a median (IQR) CD4+ T-cell count of 171 (161–201) cells/μL. All of them had HIV viral load ≤ 50 copies/mL. Two years after the PML-IRIS diagnosis, 6 (54.5%) patients were alive, and their median (IQR) CD4+ T-cell count was 237 (179–283) cells/μL, with 4 of them presenting HIV viral load ≤ 50 copies/mL. The other 2 patients had 11,812 copies/mL (case 1) and 17,183 copies/mL (case 9), respectively, but irregular use of cART was reported on their medical records.

## DISCUSSION

In this study, the prevalence of PML-IRIS among PLWHA with definite PML, assessed in the context of a tertiary reference hospital, was 11.8%. There were 4 (36%) in-hospital deaths, three being associated with hospital-acquired pneumonia. Six (54.5%) patients were alive 1 year after the PML-IRIS diagnosis.

Immune reconstitution inflammatory syndrome is a common complication of cART initiation and is associated with considerable morbidity and mortality. 15 21 Central nervous system (CNS) IRIS develops in 9 to 47% of PLWHA and is associated with a mortality of approximately 20 to 30%. 22 Frequency and outcomes of IRIS vary widely depending on the underlying infection and on individual circumstances, 22 and cryptococcal and tuberculous meningitis are the most studied. 15

Although well characterized in clinical practice, PML-IRIS has not been frequently reported, as demonstrated in some reviews about this topic. 14 15 18 Müller et al. (2010) identified 54 cohort studies published between 1996 to 2009 including 13,103 patients starting cART. Among them, 1,699 (13%) were reported to have developed IRIS, and only 2 (3.7%) of 54 patients with definite PML had IRIS. 15 Tan et al. (2009) identified 54 PLWHA patients with PML-IRIS reported in 23 articles from 1998 to 2007. 18 Fournier et al. (2017) identified 46 PLWHA patients with definite PML-IRIS reported in 31 articles published between 1998 and 2016. 14 Here, we identified a prevalence of PML-IRIS among PLWHA patients with definite PML of 11.8%. Several studies with heterogeneous criteria and design suggest that 4 to 42% of patients with HIV-related PML develop IRIS. 23

Our sample's demographic and immunological profile before cART was similar to the results of the two main reviews on PML-IRIS, which mostly included patients from high-income countries. 14 18 All of our patients were diagnosed with HIV infection before admission and most of them had a history of AIDS-defining disease, showing a current trend in the profile of hospitalized PLWHA. We identified a median time from initiation of cART to PML-IRIS diagnosis of 49 days, similar to the 35 to 53 days previously described. 14 18 Interestingly, the immunological profile at PML-IRIS diagnosis was lower in our study (median CD4 T cell count = 50 cells/μl) compared to the results of a prior review (median CD4 T cell count = 101 cells/μl). 14 Despite the limitations of this type of comparison, we can speculate that the lower immunological recovery observed in the short term in our study may be due, at least in part, to a longer period of immunosuppression before cART or to loss of follow-up. Loss of follow-up might happen because of various barriers hindering timely access to health systems, which are generally more relevant in low- and middle-income countries. Approximately 75% of our patients had unmasking PML-IRIS, similar to the 67% described in one study, 18 but considerably different from the 46% reported in another review. 14 This discrepancy is probably explained by the different inclusion criteria used in these studies. Probably, the use of less stringent diagnostic criteria tends to result in higher rates of unmasking PML-IRIS, as reported in 72% of cases in a single center study. 20

The diagnosis of IRIS in PLWHA is challenging. Several diagnostic criteria have been proposed, 15 24 25 26 but the use of other definitions or unclear criteria is common in the literature. 15 An interesting issue is the need to include increased CD4+ T-cell count as a criterion for IRIS. Shelburne et al. (2002) considered the decrease in HIV viral load from baseline or an increase in CD4+ T-cell count from baseline. 24 French et al. (2004) considered an increase in blood CD4+ T-cell count after cART as a minor criterion of IRIS. 25 These recommendations consider that decreasing HIV viral load rather than rising CD4+ T-cell counts might be a more sensitive finding of IRIS. 13 All PLWHA with PML-IRIS in this study were severely immunosuppressed, and their median CD4+ T-cell counts before cART and at PML-IRIS diagnosis were similar. A modest increase in CD4+ T-cell count at PML-IRIS onset was previously reported 14 and was comparable to the CD4+ T-cell counts observed in PML patients before the cART era. 19 Another study showed that the rise in CD4+ T-cell counts from baseline to IRIS diagnosis was not statistically significant. 27 In contrast, the rise of CD4 cell count over the first 3 months of cART was associated with IRIS. 28 This finding suggests that longer time periods of observation may be needed to detect immunological changes associated with IRIS. Accordingly, 1 year after the diagnosis of PML-IRIS, the median CD4+ T-cell count in our surviving patients was 171 cells/μL, and all of them had undetectable HIV viral load. Thus, clinicians that suspect IRIS in the first months after the initiation of cART should be more attentive to the change in HIV viral load levels rather than to the CD4+ T-cell counts when using response to therapy as a diagnostic criterion. 13

The diagnosis of PML-IRIS may be difficult since the diagnostic methods used (i.e., CSF JCV-PCR, brain MRI, consecutive HIV viral load) are expensive and not available in most resource-limited settings. 22 29 A consequence of this scenario is the scarce information available on CNS-IRIS from low- and middle-income countries. 29 30 For instance, prior to the present report, only 12 PLWHA with definite or possible PML-IRIS were reported in two studies conducted in Brazil. 31 32

In this study, we used qualitative CSF JCV PCR information since this is the only data available in our assistance activities. In the cART era, a significant reduction in the diagnostic positive detection rate and in the predictive value of the negative test were observed. 33 The immune reconstitution, represented by CD4 count above 100 cells/μl was demonstrated to be an independent predictor of failure to detect JCV DNA in the CSF of PML patients. 33 In this context, monitoring the quantitative CSF JCV-PCR testing over time could be an indicator of PML-IRIS.

Despite the absence of controlled randomized trials demonstrating the benefit of corticosteroids in the treatment of CNS-IRIS, the fact that only observational evidence is available, 22 29 34 and the controversies about its use, 35 36 all but one of the patients of our sample received corticosteroids.

Survival of PLWHA patients with PML has improved in recent years. In the pre-cART era, only 10 to 35% of PLWHA patients were alive 1 year after the diagnosis of PML. In the cART era, the 1-year survival rate has increased to 50 to 70%. 10 37 38 39 40 41 However, this increase in PML survival is smaller than the one of more frequent opportunistic diseases. 38 In-hospital mortality was high (36%) in our study and 3 of 4 cases were secondary to in-hospital pneumonia. In addition, survival 1 and 2 years after the PML-IRIS diagnosis was 54.5%. In contrast to our outcomes, a systematic review including 43 PLWHA with PML-IRIS mostly from high-income countries and followed for a median of 8 months reported that 72% of cases improved or stabilized within 2 years. 14 Similarly, a study conduct in Spain including 18 PLWHA with PML-IRIS reported a PML a mortality attributed to PML of 22%. 20 Different of these two studies, another review including 54 PLWHA with PML-IRIS mostly living in high-income countries reported a 1-year survival of 55.6%, 18 very similar to our results. Therefore, it is currently unknown whether the long-term outcomes of PLWHA with PML-IRIS are better in high-income countries than in middle-income countries, like Brazil.

Despite the improvement in survival observed in the cART era, PML continues to cause high morbidity. For example, a nationwide study reported 52% of progression of neurological symptoms or unchanged neurological symptoms in PLWHA with PML after four months of follow-up. 11 We identified a higher rate (71.5%) of neurological sequelae at hospital discharge, suggesting an additional concern regarding patients who survive PML-IRIS.

In conclusion, the prevalence of PML-IRIS among PLWHA with definite PML was 11.8%. Clinical and laboratory baseline findings were similar to prior reports. However, the median CD4+ T-cell count was low at PML-IRIS diagnosis suggesting the relative value of this parameter in the definition of IRIS. The in-hospital mortality was high and all but one death were due to hospital-acquired pneumonia. The 1-year survival was similar to the one described in some studies performed in high-income countries.
